# Liposome-Based Encapsulation of Extract from Wild Thyme (*Thymus serpyllum* L.) Tea Processing Residues for Delivery of Polyphenols

**DOI:** 10.3390/foods14152626

**Published:** 2025-07-26

**Authors:** Aleksandra A. Jovanović, Bojana Balanč, Predrag M. Petrović, Natalija Čutović, Smilja B. Marković, Verica B. Djordjević, Branko M. Bugarski

**Affiliations:** 1 Institute for the Application of Nuclear Energy, University of Belgrade, Banatska 31b, 11080 Belgrade, Serbia; 2 Innovation Centre of the Faculty of Technology and Metallurgy, University of Belgrade, Karnegijeva 4, 11000 Belgrade, Serbia; bisailovic@tmf.bg.ac.rs (B.B.);; 3 Institute for Medicinal Plants Research “Dr. Josif Pančić”, Tadeuša Košćuška 1, 11000 Belgrade, Serbia; ncutovic@mocbilja.rs; 4 Institute of Technical Sciences of SASA, Knez Mihailova 35/IV, 11000 Belgrade, Serbia; smilja.markovic@itn.sanu.ac.rs; 5 Faculty of Technology and Metallurgy, University of Belgrade, Karnegijeva 4, 11000 Belgrade, Serbia; vmanojlovic@tmf.bg.ac.rs (V.B.D.); branko@tmf.bg.ac.rs (B.M.B.)

**Keywords:** β-sitosterol, cholesterol, liposomes, proliposome method, *Thymus serpyllum*

## Abstract

This study developed phospholipid-based liposomes loaded with extract from wild thyme (*Thymus serpyllum* L.) tea processing residues to enhance polyphenol stability and delivery. Liposomes were prepared with phospholipids alone or combined with 10–30 mol% cholesterol or β-sitosterol. The effect of different lipid compositions on encapsulation efficiency (EE), particle size, polydispersity index (PDI), zeta potential, stability, thermal properties, diffusion coefficient, and diffusion resistance of the liposomes was investigated. Liposomes with 10 mol% sterols (either cholesterol or β-sitosterol) exhibited the highest EE of polyphenols, while increasing sterol content to 30 mol% resulted in decreased EE. Particle size and PDI increased with sterol content, while liposomes prepared without sterols showed the smallest vesicle size. Encapsulation of the extract led to smaller liposomal diameters and slight increases in PDI values. Zeta potential measurements revealed that sterol incorporation enhanced the surface charge and stability of liposomes, with β-sitosterol showing the most pronounced effect. Stability testing demonstrated minimal changes in size, PDI, and zeta potential during storage. UV irradiation and lyophilization processes did not cause significant polyphenol leakage, although lyophilization slightly increased particle size and PDI. Differential scanning calorimetry revealed that polyphenols and sterols modified the lipid membrane transitions, indicating interactions between extract components and the liposomal bilayer. FT-IR spectra confirmed successful integration of the extract into the liposomes, while UV exposure did not significantly alter the spectral features. Thiobarbituric acid reactive substances (TBARS) assay demonstrated the extract’s efficacy in mitigating lipid peroxidation under UV-induced oxidative stress. In contrast, liposomes enriched with sterols showed enhanced peroxidation. Polyphenol diffusion studies showed that encapsulation significantly delayed release, particularly in sterol-containing liposomes. Release assays in simulated gastric and intestinal fluids confirmed controlled, pH-dependent polyphenol delivery, with slightly better retention in β-sitosterol-enriched systems. These findings support the use of β-sitosterol- and cholesterol-enriched liposomes as stable carriers for polyphenolic compounds from wild thyme extract, as bioactive antioxidants, for food and nutraceutical applications.

## 1. Introduction

Leftover plant material from the production of tea bags made from *Thymus serpyllum* L. (wild thyme, Lamiaceae) consists of small particles, dust, broken leaves, and stems that are either too fine or too coarse to be separated effectively. These residues are generally excluded from the final tea bags, as their presence can compromise both brewing quality and the structural integrity of the bags. Nevertheless, this solid plant waste from wild thyme still contains valuable bioactive compounds, including essential oil, polyphenols (flavonoids, phenolic acids, and anthocyanins), monoterpenes, polysaccharides, and proteins [1,2]. Extracts obtained from the industrial processing of plant materials hold promising potential for use in food, pharmaceutical, and cosmetic applications. For instance, polyphenol-rich extracts from grape and olive pomace, common food industry by-products (waste), may be directly applicable in these industries [3,4]. This approach eliminates the need for labor-intensive and costly downstream purification and isolation procedures. Our previous study revealed that microwave-assisted extraction can be considered an effective method for recovering polyphenols from *T. serpyllum* by-product [2]. Polyphenol compounds are widely used in various sectors of the food-processing industry as natural additives, such as antioxidants and nutritional, coloring, and preservative agents. Furthermore, various plant extracts rich in polyphenols are used as ingredients within pharmaceutical or cosmetic formulations [5]. However, the utilization of these valuable natural compounds is rather limited due to their low bioavailability, integrity, permeability, and solubility. Specifically, the inherent instability of polyphenols and their susceptibility to environmental factors during food processing, distribution, storage, as well as varying conditions in the gastrointestinal tract (including pH fluctuations, enzymatic activity, and interactions with other nutrients), significantly limit their bioactivity and potential health benefits. Additionally, many polyphenol compounds have an unpleasant taste that restricts their incorporation into foodstuffs or drugs intended for oral administration [6]. Thus, encapsulation, as a technique to incorporate active agents into particles, represents an appropriate way to overcome the disadvantages [7,8]. Fluid bed coating, melt injection, melt extrusion, emulsification, coacervation, inclusion complexation, liposome entrapment, freeze drying, spray drying, or vacuum drying are commonly used encapsulation processes [9]. Namely, the encapsulation of active compounds in liposomes can be advantageous for several reasons. The hydrophilic, lipophilic, or amphiphilic substances encapsulated in liposomes are formulated as a solution, exhibiting higher bioavailability. Having in mind that liposomes form spontaneously upon phospholipid hydration, they achieve good physical stability during storage. Depending on the production method, liposomal particles can attain nanoscale diameters, which contributes to the formation of water-dispersible formulations exhibiting near-transparent clarity [10]. Moreover, cholesterol plays a crucial role within the phospholipid bilayer by modulating membrane characteristics such as fluidity, permeability, and structural integrity. This is achieved through its interaction with both the hydrophobic fatty acid chains and the hydrophilic phosphate headgroups of phospholipids [11]. Specifically, the hydrophobic segment of cholesterol aligns with the fatty acid tails inside the membrane core, while its polar hydroxyl group associates with the membrane surface near the phospholipid head regions [12]. As a result, cholesterol contributes to the organization and stability of the bilayer structure and influences the physical properties of liposomal membranes. Additionally, variations in the phospholipid-to-cholesterol ratio affect membrane rigidity, mechanical strength, and selective permeability [11]. Further, β-sitosterol is recognized as the most abundant phytosterol, structurally similar to cholesterol but distinguished by an additional ethylene group at the C-24 position. It exhibits a range of beneficial biological activities, including anti-inflammatory, anti-diabetic, anti-cancer, immunomodulatory, and cholesterol-lowering effects [13,14]. Studies have shown that increasing the content of β-sitosterol within phospholipid liposome bilayers results in greater membrane rigidity and decreased permeability [14]. Research by Lee et al. [13] demonstrated that β-sitosterol, due to its favorable physiological properties, can enhance the stability of bioactive compounds encapsulated in liposomal systems, highlighting its potential application in liposomal encapsulation technologies.

In the present study, liposomes containing pure phospholipids (Ph), Ph-cholesterol, and Ph-β-sitosterol (as the carrier for *T. serpyllum* by-product extract) were developed. Specifically, the effect of cholesterol and β-sitosterol on encapsulation efficiency (EE), particle size, polydispersity index (PDI), zeta potential, stability, antioxidant properties, and thermal characteristics of the liposomes was investigated. An in vitro release behavior in water and simulated gastric and intestinal fluids was also examined.

## 2. Materials and Methods

### 2.1. Materials

The by-product of wild thyme herb (i.e., tea processing residues according to the Regulations on the quality of tea, herbal tea, and their products of the Republic of Serbia [15]) was obtained from the Institute for Medicinal Plants Research “Dr. Josif Pančić”, Belgrade, Serbia. The following reagents were used: ethanol and sodium carbonate (Fisher Scientific, Loughborough, UK), Folin-Ciocalteu reagent and gallic acid (Merck, Darmstadt, Germany), Phospholipon 90 G (granulated, purified soy lecithin product, primarily composed of phosphatidylcholine, with a content of at least 94.0%) (Lipoid GmbH, Ludwigshafen am Rhein, Germany), cholesterol (Croda, Goole, UK), and β-sitosterol (Sigma-Aldrich, St. Louis, MA, USA).

### 2.2. Extract Preparation

By-product *T. serpyllum* liquid extract was prepared using 48% ethanol as the extraction solvent and a solid-to-solvent ratio of 40.2 mg/mL in microwave-assisted extraction, using the microwave reactor (Monowave 300, Anton Paar, Graz, Austria), according to optimized conditions in a previously published study [2].

### 2.3. Liposomes Preparation

Wild thyme extract was encapsulated in the following liposomal systems: liposomes containing 100% of phospholipids (Ph), 90 mol% of Ph and 10 mol% of sterol, i.e., cholesterol or β-sitosterol (Ph+chol 10% and Ph+β-sito 10%, respectively), 80 mol% of Ph and 20 mol% of sterol (Ph+chol 20% and Ph+β-sito 20%), and 70 mol% of Ph and 30 mol% of sterol (Ph+chol 30%, and Ph+β-sito 30%).

Wild thyme by-product extract-loaded liposomes were prepared using the proliposome method [16]. Liposomes were formed using pure Ph or the mixture of Ph and sterol (10, 20, and 30 mol% of cholesterol or β-sitosterol). Namely, a mixture of lipids, i.e., cholesterol or β-sitosterol and commercial phospholipid mixture (ratio depended on the used mol% of sterols), in the total amount of 1 g and liquid *T. serpyllum* ethanol extract in the volume of 4 mL were added in a glass beaker and stirred at 50 °C for 30 min at a speed of 800 rpm using a stir bar (small white oblong Teflon-covered magnet) in the uncovered beaker on the magnetic stirrer (RET basic, IKA, Staufen, Germany). Due to the presence of ethanol (from the liquid extract) and high temperature, phospholipids and sterols were dissolved, and a molten mixture was obtained. After complete homogenization of the sample and evaporation of ethanol (due to the elevated temperature and uncovered beaker), the mixture was cooled to room temperature, and the aqueous phase (ultrapure water, 20 mL) was added in small portions. With the aim of developing liposomal vesicles, the proliposome mixture converts to a liposomal dispersion by the slow addition of water. The dispersion was continuously stirred at 800 rpm and 25 °C for 1 h, in a covered beaker on a magnetic stirrer. Empty liposomes were prepared as a control using the same protocol (without the addition of wild thyme extract).

### 2.4. Encapsulation Efficiency Analysis

EE was determined using an indirect method. EE was calculated by the amount of polyphenols in the supernatant as shown in Equation (1):

(1) $$EE[\%]=({TPC}_{i}-{TPC}_{sup})/{TPC}_{i}\times 100$$

where TPC_i_ is the initial content of total polyphenols used for the preparation of liposomes, and TPC_sup_ is the content of total polyphenols determined in the supernatant.

Free wild thyme extract was removed from liposome dispersions by centrifugation at 17,500 rpm and 4 °C for 45 min in a Thermo Scientific Sorval WX Ultra series ultracentrifuge (ThermoScientific, Waltham, MA, USA). Total polyphenol content (TPC) in extract and supernatants was determined spectrophotometrically at 765 nm (UV-1800, Shimadzu, Kyoto, Japan) using the modified Folin-Ciocalteu method [17].

### 2.5. Size, PDI, and Zeta Potential Analysis

The size, PDI, and zeta potential of liposomes were determined by photon correlation spectroscopy (PCS) in Zetasizer Nano Series, Nano ZS (Malvern Instruments Ltd., Malvern, UK). Each sample (diluted 500 times) was measured three times at room temperature.

### 2.6. Stability Study

The measurements of liposome particle size, PDI, and zeta potential were repeated on the 1st, 7th, 14th, 21st, 28th, and 60th day after preparation of suspensions to define the stability of the liposomes (empty and extract-loaded liposomes). The liposome suspensions were stored in the refrigerator at 4 °C. Moreover, a UV-stability study was also performed; a laminar flow cabinet (AC2-4G8, ESCo, Singapore) was used for the irradiation experiment. Empty and extract-loaded liposomes (3 mL) in uncovered Petri dishes were exposed to UV-C irradiation (253.7 nm) at room temperature for 20 min; subsequently, size, PDI, zeta potential, and TPC measurements were performed. Furthermore, the influence of the freeze-drying process on physical stability and TPC in liposomes was investigated after lyophilization in Beta 2-8 LD plus (Christ, Osterode am Harz, Germany). First, freshly prepared empty and extract-loaded liposomes (5 mL) were centrifuged; the supernatant was discarded, and the pellet was dispersed in 2 mL of water. The samples were frozen at −80 °C for 1 h using a LAB11/EL19LT freezer (Elcold, Hobro, Denmark), followed by freeze-drying at −75 °C and 0.011 mbar for 24 h. The lyophilized liposomes were subsequently reconstituted with ultrapure water to their original volume in safe-lock plastic tubes (2 mL) and stirred using a lab dancer test tube shaker (IKA, Staufen, Germany) for 2 min before further analysis (PCS and TPC analyses). The cryoprotectants were not used, with the aim of investigating the influence of the freeze-drying process on the characteristics of developed liposomes without protective agents.

### 2.7. Differential Scanning Calorimetry

Thermal properties of selected empty and extract-loaded liposomes (Ph, Ph+chol 10%, and Ph+β-sito 10%), as well as of pure Phospholipon and extract, were analyzed in DSC131 Evo (SETARAM Instrumentation, Caluire-et-Cuire, France). Due to the specific operational constraints of the device, only dried and/or lyophilized liposomal samples were compatible with the testing protocol. The samples were placed in small aluminum containers (30 μL), which were subsequently hermetically sealed. Before analysis, the device was calibrated using indium, with an empty container serving as the reference standard. First, both aluminum containers (reference and sample) were thermostated at 30 °C for 5 min. The phase transition changes were monitored from 30 °C to 300 °C, with a heating rate of 10 °C/min; the nitrogen flow was 20 mL/min. A baseline run was performed using empty containers under the same conditions; the baseline subtraction and determination of enthalpy (J/g) were carried out using CALISTO PROCESSING software (version 2.16) equipped with SETARAM Instrumentation.

### 2.8. Fourier Transform Infrared Spectroscopy

FT-IR spectra of the lyophilized *T. serpyllum* extract, selected empty and extract-loaded liposomes (Ph, Ph+chol 10%, and Ph+β-sito 10%, non-treated and UV-irradiated samples), as well as of pure Phospholipon, cholesterol, and β-sitosterol, were recorded in the transmission mode between 400 and 4000 cm^−1^ using a Nicolet iS10 spectrometer (Thermo Scientific, Stockholm, Sweden). Due to the device’s operational parameters, only lyophilized (dried) samples were amenable to testing.

### 2.9. Thiobarbituric Acid Reacting Substances Assay

The peroxidation of selected liposomal populations (Ph, Ph+chol 10%, and Ph+β-sito 10%) with wild thyme extract was tested using the thiobarbituric acid-reacting substances (TBARS) method. Empty liposomes were used as a control. Plain and extract-loaded liposomes were exposed to UV light irradiation for 12 h. Control samples were also taken from the same batch and stored in the dark at ambient temperature. At certain time intervals during the 12 h period, 100 μL of sample was taken [18]. In short, the liposomes were mixed with 1.5 mL of 20% trichloroacetic acid solution and 1 mL of the stock solution containing 2% thiobarbituric acid and 20% perchloric acid in a 1:1 ratio, and heated at 100 °C. The samples were cooled to stop the reaction (at 25th min) and centrifuged at 3000 rpm for 8 min to eliminate precipitate. The absorbance of the pink color of the supernatant, from the reaction between lipid hydroperoxide and thiobarbituric acid, was measured spectrometrically at 532 nm.

### 2.10. In Vitro Release Study

An in vitro release study was performed using a Franz diffusion cell (donation of PermeGear, Inc., Hellertown, PA, USA) with two compartments separated by an acetate-cellulose membrane (pore size of 0.2 μm). The study included the selected extract-loaded liposomes (the samples with the highest EE in each liposome group—Ph, Ph+chol 10%, and Ph+β-sito 10%), as well as pure extract. The sample was placed in the donor compartment (1.5 mL), while the receptor compartment was filled with water and constantly mixed at 360 rpm using a magnetic stirrer (at 25 °C). The release of polyphenols was monitored over 24 h, with samples collected from the receptor compartment at predefined time intervals. The concentration of polyphenols in the samples was determined spectrophotometrically at 280 nm. The in vitro release study of polyphenols from the pure extract and selected extract-loaded liposomal samples (Ph, Ph+chol 10%, and Ph+β-sito 10%) in simulated gastrointestinal fluids was also performed at 37 °C. The only difference was the simulated gastrointestinal fluids in the receptor compartment of the diffusion cell instead of water. Simulated gastric fluid (SGF) and simulated intestinal fluid (SIF) were prepared according to Trifković et al. [8]. The pH value of SGF was adjusted to 2.2 using hydrochloric acid, while the pH value of SIF was adjusted to 6.8 using sodium hydroxide.

### 2.11. Statistical Analysis

In the present study, statistical analysis was performed using analysis of variance (one-way ANOVA) followed by Duncan’s post hoc test within the statistical software, STATISTICA 7.0. The differences were considered statistically significant at *p* < 0.05.

## 3. Results and Discussion

### 3.1. Encapsulation Efficiency

EE of polyphenols in different liposomal systems is shown in Table 1 (the values obtained immediately after the liposomal preparation, UV irradiation, and lyophilization).

As can be seen from Table 1, liposomes containing 10 mol% of cholesterol or β-sitosterol showed the highest EE of polyphenols from wild thyme extract, followed by liposomes with 20 mol% of sterols and with pure phospholipids (without sterols). Liposomes containing 30 mol% cholesterol or β-sitosterol exhibited the lowest EE. Compared to Ph liposomes, samples with cholesterol or β-sitosterol showed slightly higher EE, which can be attributed to the increased hydrophobicity and enhanced stability imparted by sterols, resulting in more efficient entrapment of the active compound. On the other hand, higher concentrations of sterols in formulations may compete with active compounds for packing space, causing lower EE [19]. In addition, the explanation of the result can lie in an increase in the fluidity (permeability) of the liposomal membrane caused by a higher concentration of sterols, which leads to an increase in the fluidity (permeability) of the liposomal membrane. Due to the insertion of sterols between phospholipid molecules in liposomes, there is an increase in membrane permeability, which facilitates the release of polyphenols from the liposomal carrier and therefore causes reduced encapsulation of active components [7].

### 3.2. Liposomes Size, PDI, and Zeta Potential

**Figure 1:**
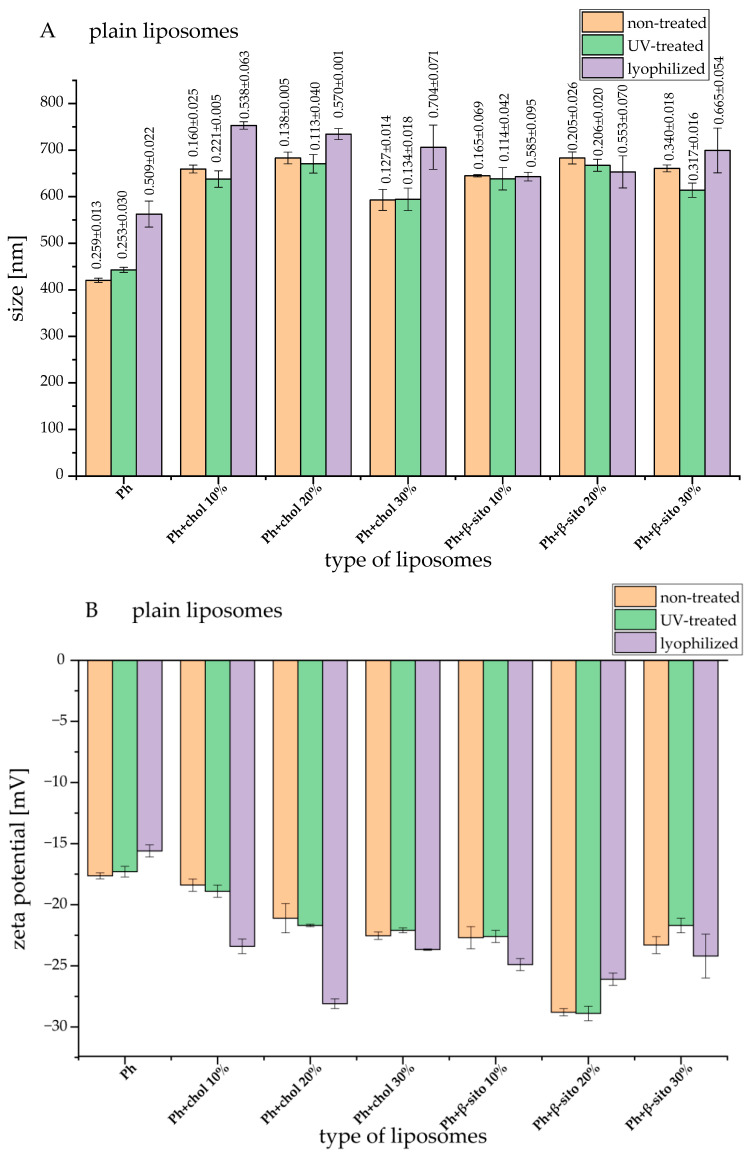

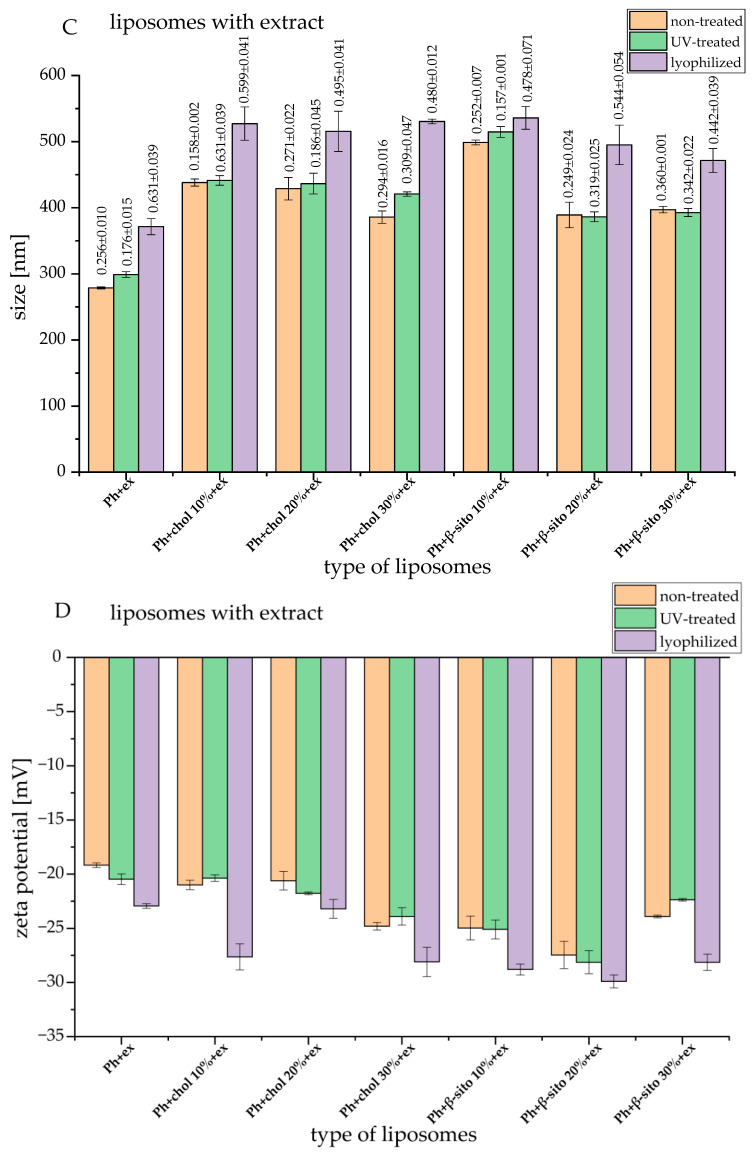
Particle size—bars and polydispersity index—numbers above bars (**A**,**C**) and zeta potential (**B**,**D**) of plain and wild thyme extract-loaded liposomes, respectively, obtained immediately after the liposomal preparation, UV irradiation, and lyophilization; liposomes containing 100% phospholipids (Ph), liposomes containing 90–70 mol% of Ph and 10–30 mol% of sterol, i.e., cholesterol or β-sitosterol (Ph+chol and Ph+β-sito, respectively); ex, extract.

The influence of different lipid composition and sterol amounts on particle size, PDI, and zeta potential of empty liposomes and wild thyme extract-loaded liposomes was examined using PCS; the results (the values obtained immediately after the liposomal preparation, UV irradiation, and lyophilization) are shown in Figure 1.

As can be seen in Figure 1A,C, pure phospholipids have given the smallest liposomal particles, and the addition of cholesterol or β-sitosterol led to an increase in liposomal particle size. The increase in particle size due to the presence of sterols is attributed to the interactions between lipid chains in the area of the phospholipid head, inter-lipid space formation, and expansion of the liposomal membrane [20,21]. In the case of all samples, the liposomes with extract had a smaller diameter than their empty counterparts. Namely, the encapsulation of the extract within the liposomal membrane reduces the number of lipid and sterol molecules that are incorporated into the membrane and, therefore, causes the formation of smaller vesicles [22]. Tighter lipid organization may result from the extract’s bioactive ingredients integrating into the lipid bilayer. By restricting the flexibility and curvature of the membrane, this higher molecular packing may result in the production of smaller vesicles. Additionally, the presence of ethanol residues in the formulations (due to the ethanol *T. serpyllum* extract) also affects the particle size. There is a theory that ethanol causes a change in the net charge in the liposomal system and, thus, provides steric stabilization in part [23]. This may be the reason for the smaller average diameter of liposomes with encapsulated extract.

Since liposomal dispersion can possess a polydisperse character depending on the applied preparation technique and composition, it is important to determine the PDI values as a measure of particle size distribution. As can be seen in Figure 1A,C (numbers above bars), empty liposomes had PDI values between 0.127 and 0.340 (presented as the values above bars in Figure 1A), and the liposomes with extract had PDI values between 0.182 and 0.360 (presented as the values above bars in Figure 1C). In both cases, liposomes with 30 mol% of β-sitosterol possessed the highest PDI. Namely, according to the literature data, higher concentrations of cholesterol and β-sitosterol led to higher PDI values, showing the increased heterogeneity [21]. Furthermore, the addition of the extract to the liposomes results in a slight increase in the polydispersity of the system. According to the literature data, greater dispersions were produced by a higher loading of plant-based bioactives in liposomal spheres, and PDI values increased as the extract content encapsulated in the liposomal vesicles increased [24]. However, all obtained PDI values indicate the existence of a uniform or moderately uniform system within the liposomal suspension.

The values of zeta potential refer to the total charge that exists on the surface of the vesicles in the dispersion medium and therefore testify to the stability of the system [7]. Particles are considered stable if the zeta potential is greater than +30 mV or less than −30 mV [21,25]. The zeta potential values of empty liposomes and liposomes with extract are shown in Figure 1B,D. Liposomes containing sterol showed higher zeta potential (absolute value) compared to pure phospholipid liposomes, regardless of sterol type and concentration. However, liposomes containing β-sitosterol had a statistically significantly higher absolute value of zeta potential than liposomes with pure phospholipids and liposomes with cholesterol. According to the literature, incorporating sterols increases the spacing between phospholipid head groups and enhances hydrophobic stabilization of bilayer membranes [20,26,27]. Moreover, the presence of sterols can change the order of phospholipids and the thickness of the bilayer, regardless of the nature of the functional groups in phospholipids, and these groups can participate in creating hydrogen bonds with sterols, as well as in changing the total charge [26,28]. The encapsulation of wild thyme extract did not cause a statistically significant change in the zeta potential of liposomes. According to our previous study, the encapsulation of gentisic acid in β-sitosterol liposomes also did not affect the value of zeta potential [29].

### 3.3. The Stability of Liposomal Vesicles

With the aim of examining the stability of the obtained liposomal systems (empty liposomes and liposomes containing wild thyme extract), particle size, PDI, and zeta potential were measured for 60 days. The particle sizes of all liposomes did not change drastically during 60 days of storage in refrigeration (Figure S1A,B, Supplementary Materials). Particle size with pure phospholipids (without sterols) during the 60-day stability study varied between ~420 nm and ~580 nm (empty liposomes) and between ~230 nm and ~360 nm (liposomes with extract). Liposomes containing cholesterol had a diameter from ~550 nm to ~690 nm (empty liposomes) and from ~360 nm to ~460 nm (liposomes with extract). Liposomes with β-sitosterol had a diameter between ~600 nm and ~690 nm (empty liposomes) and between ~350 nm and ~500 nm (liposomes with extract). Throughout the 60-day storage period, all liposomal formulations exhibited moderate fluctuations in particle size, with sterol-containing liposomes generally maintaining greater size stability compared to those composed solely of phospholipids. The mentioned observation can be related to the previously shown higher zeta potential (absolute value) of liposomes containing sterols in comparison to pure phospholipid liposomes (Figure 1B,D). In all samples, PDI values showed no significant change throughout the 60-day period of storage in refrigeration (Figure S1C,D). On the other hand, during the 60-day stability study, the zeta potential varied in almost all samples, but the trend depended on the composition of the liposomal membrane (Figure S1E,F). In the empty Ph liposomes, zeta potential did not vary for 60 days, whereas in the Ph liposomes with extract, zeta potential (absolute value) increased until the 21st day, from −19.2 ± 0.2 mV to −24.7 ± 1.0 mV. In the empty cholesterol liposomes, zeta potential varied between −18.0 ± 0.3 mV and −22.5 ± 0.7 mV, and in the liposomes containing cholesterol and extract, zeta potential varied from −19.0 ± 0.2 mV to −24.8 ± 0.3 mV. In empty β-sitosterol liposomes, zeta potential was between −18.3 ± 0.4 mV and −28.8 ± 0.3 mV, while liposomal vesicles containing β-sitosterol and extract had zeta potential from −20.1 ± 0.7 mV to −27.5 ± 1.3 mV. The observed changes in zeta potential during the 60-day storage period are likely due to multiple, interconnected factors influencing the stability of surface charge in liposomal systems. One contributing mechanism may be the structural reorganization or oxidative degradation of phospholipids, which can expose or reposition charged headgroups, thereby altering surface electrostatics [30]. Additionally, the slow diffusion or release of encapsulated bioactives may lead to interactions at the vesicle interface or in the surrounding medium, potentially affecting net charge. Liposome fusion or aggregation during storage also reduces the overall surface area and redistributes charges, both of which influence measured values [31]. The presence of sterols (cholesterol or β-sitosterol) can further modulate these effects, either enhancing membrane rigidity and stability or, conversely, altering the lipid arrangement in ways that affect surface charge characteristics [32].

Since UV irradiation is applied in food, pharmaceutical, and cosmetic industries, the effect of 20-min UV irradiation on the particle size, PDI, and zeta potential (Figure 1) and EE of polyphenols was examined (Table 1). Comparing the values obtained immediately after liposome preparation and UV irradiation, it can be concluded that the treatment did not cause changes in particle size, uniformity (PDI), stability (zeta potential), or polyphenol leakage in the system. However, according to the literature data, an increase in liposome diameter following UV exposure has been reported in prior studies, indicating that photochemical degradation may occur due to photon energy absorption, which substantially alters the structural organization of the bilayer membrane. UV irradiation disrupts the arrangement and packing of phospholipid molecules, resulting in significant changes to their physical properties, including increased vesicle size and altered membrane permeability [33,34]. In addition, the application of energy, including thermal or photonic input, to liposomal systems could induce vesicle enlargement, a reduction in zeta potential, and even gel formation [35]. Nevertheless, in the case of all plain and wild thyme extract-loaded liposomes, the mentioned alterations did not occur, probably due to the short exposure time. Ribovski et al. [36] reported that the liposomes that were subjected to UV treatment did not display any release of encapsulated compound calcein, indicating that the liposomal bilayer integrity was not compromised in the presence of the high energetic irradiation. Although UV-induced local increase in temperature or molecular changes in the liposomal membrane, the mentioned alterations did not induce the compound release. Still, upon increasing the UV exposure period, an increment in the release of calcein was noticed. The same study also showed that local heating can facilitate slight permeabilization of the lipid membrane, but UV-irradiation did not alter the particle size [36]. Additionally, the encapsulation of wild thyme bioactives within liposomal membranes may enhance structural stability and mitigate degradation caused by UV exposure. This protective effect is likely due to its integration between adjacent, loosely packed mono- and polyunsaturated phospholipid chains within the bilayer [34]. Namely, hydrophobic polyphenols can become embedded among phospholipid monolayers, effectively reinforcing the membrane and limiting further structural disruption.

Since the lyophilization process facilitates prolonged storage of products through the removal of water, while preventing microbiological contamination, the influence of the drying process on empty liposomes and liposomes with extract was studied. The results of particle size, PDI, and zeta potential are presented in Figure 1, while the EE is presented in Table 1. Comparing the values obtained immediately after liposome preparation and lyophilization, it is noticed that the freeze-drying process led to a statistically significant increase in the particle size (5–25%, except for empty β-sitosterol liposomes, where there were no changes) and PDI; the changes in zeta potential values depended on lipid/sterol composition. The observed changes in liposome diameter following lyophilization were expected, as literature data indicate that the lyophilization process can promote membrane contact and lead to the formation of larger structures through vesicle fusion or aggregation [37,38]. This effect is primarily attributed to the increased local concentration of liposomes caused by the advancing ice front, along with the loss of the hydration layer that normally inhibits membrane fusion [37]. However, lyophilization did not cause the leakage of polyphenols from liposomes.

### 3.4. Thermotropic Properties of Liposomes

**Figure 2:**
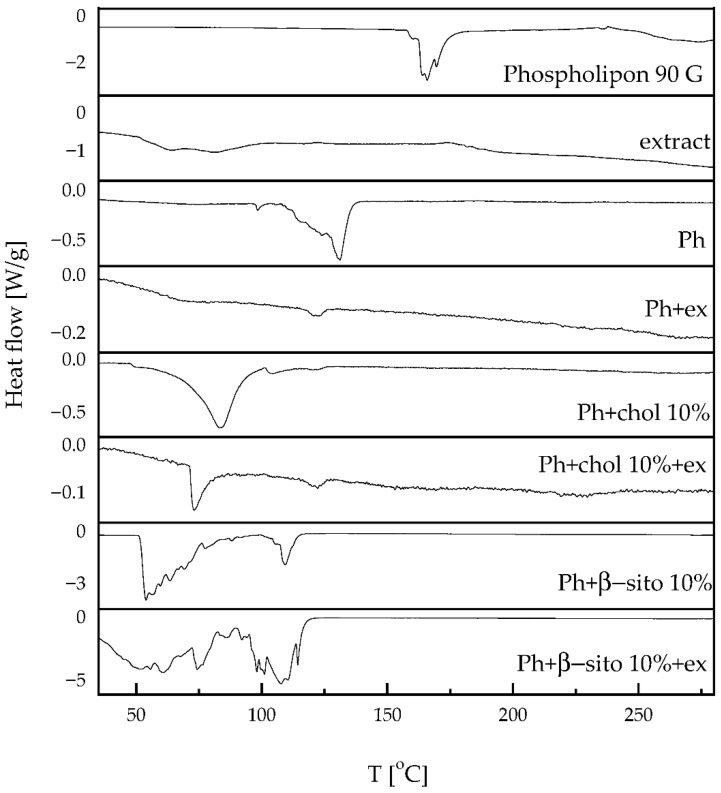
DSC thermogram of pure Phospholipon (commercial mixture of phospholipids used for the liposome preparation), lyophilized wild thyme extract, and lyophilized plain and extract-loaded liposomes; liposomes containing 100% phospholipids (Ph), liposomes containing 90 mol% of Ph and 10 mol% of sterol, i.e., cholesterol or β-sitosterol (Ph+chol 10% and Ph+β-sito 10%, respectively); ex, extract.

DSC was employed to investigate the thermal behavior of developed liposomes. Figure 2 presents the DSC thermogram of pure phospholipon, extract, as well as liposomes, both empty and loaded with the extract. The enthalpy changes are given in Table 2.

Phospholipon exhibits an endothermic peak at 165.8 °C (−110.1 J/g), most likely associated with a phase transition from the gel to the liquid-crystalline state. Although a range for the gel-to-liquid crystalline phase transition of hydrogenated soybean phosphatidylcholine was ~55 °C (for hydrated systems) [39], the occurrence of an endothermic peak at significantly higher temperatures can be explained by several factors. Namely, in the case of dry (non-hydrated) phospholipids (as stated in the present study DSC analysis), higher temperature melting transitions can be obtained, and crystalline phospholipids or polymorphic forms can melt at temperatures >150 °C [40]. In addition, phospholipids can start to degrade at ~160–180 °C [40,41], while the potential presence of other components, including residual solvents or excipients, can significantly affect this parameter. Specifically, the endothermic peak might correspond to the melting or transition of another compound (impurities) as well. The studies of Kenechukwu et al. [42] and Patil et al. [43] also showed DSC thermograms of Phospholipon 90G with endothermic peaks at higher temperatures (131 °C and 193.20 °C, respectively). It is well known that the non-polar hydrocarbon tails of phospholipids can produce a sharp endothermic peak during melting, and peaks observed at higher temperatures are typically attributed to degradation processes [44,45].

The extract displayed a broad endothermic peak centered at ~63.6 °C (−65.1 J/g), likely corresponding to the loss of volatile components from the sample. Another endothermic event is observed above 170 °C, displaying a markedly broad signal, suggesting thermal decomposition. This behavior may be attributed to the presence of various compounds in the extract, particularly phenolic compounds.

Plain liposomes exhibit a sharp endothermic peak at 131.1 °C (42.7 J/g), whereas extract-loaded liposomes display a broad, low-intensity endothermic peak at 122.4 °C (0.9 J/g). This shift indicates that the extract causes a displacement of the sharp Phospholipon peak toward lower temperatures, significantly affecting the enthalpy. Active molecules, such as polyphenols, may destabilize the lipid bilayer by acting as spacers, thereby reducing the gel-to-liquid crystalline phase transition temperature [18,44].

This effect of the extract is also evident in liposomal formulations containing cholesterol or β-sitosterol. Sterols are known to decrease the main transition temperature (T_m_) and lead to the disappearance of the pre-transition (T_pre_). Several studies have explained this phenomenon, emphasizing that the incorporation of sterols into lipid membranes induces a more ordered arrangement of the acyl chains in phospholipids [28,46,47]. According to the Farkas et al. study [48], β-sitosterol exhibits a pronounced ability to modify the molecular organization of soybean lecithin bilayers, demonstrating a stronger impact than that of cholesterol.

### 3.5. FT-IR Spectra of the Samples

**Figure 3:**
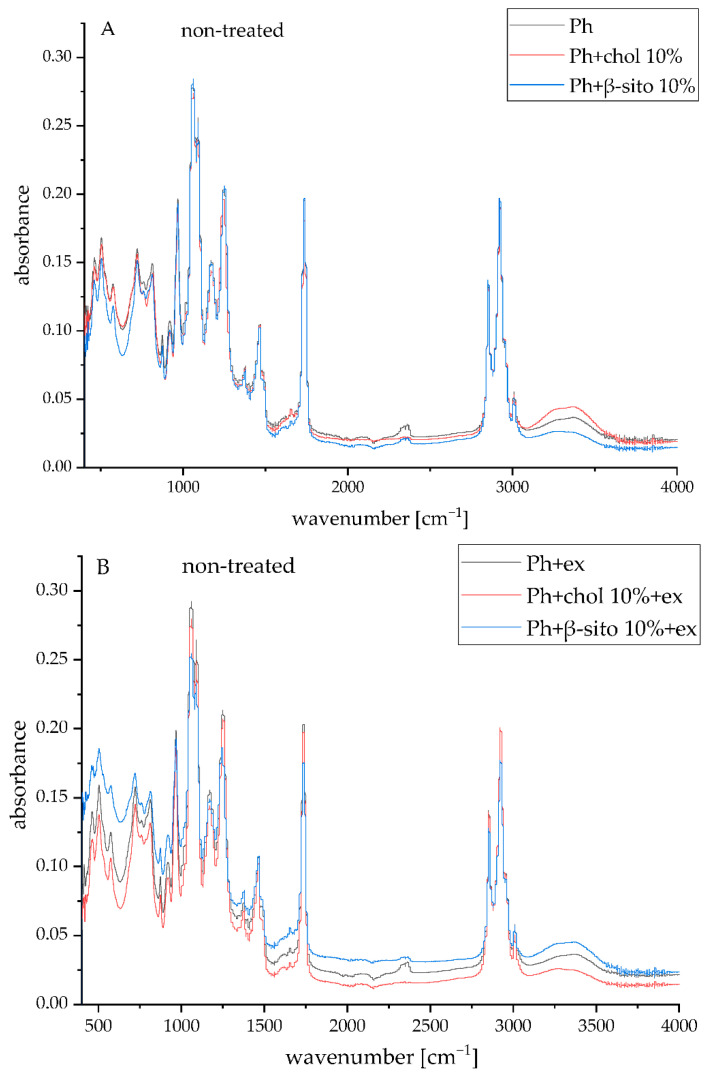

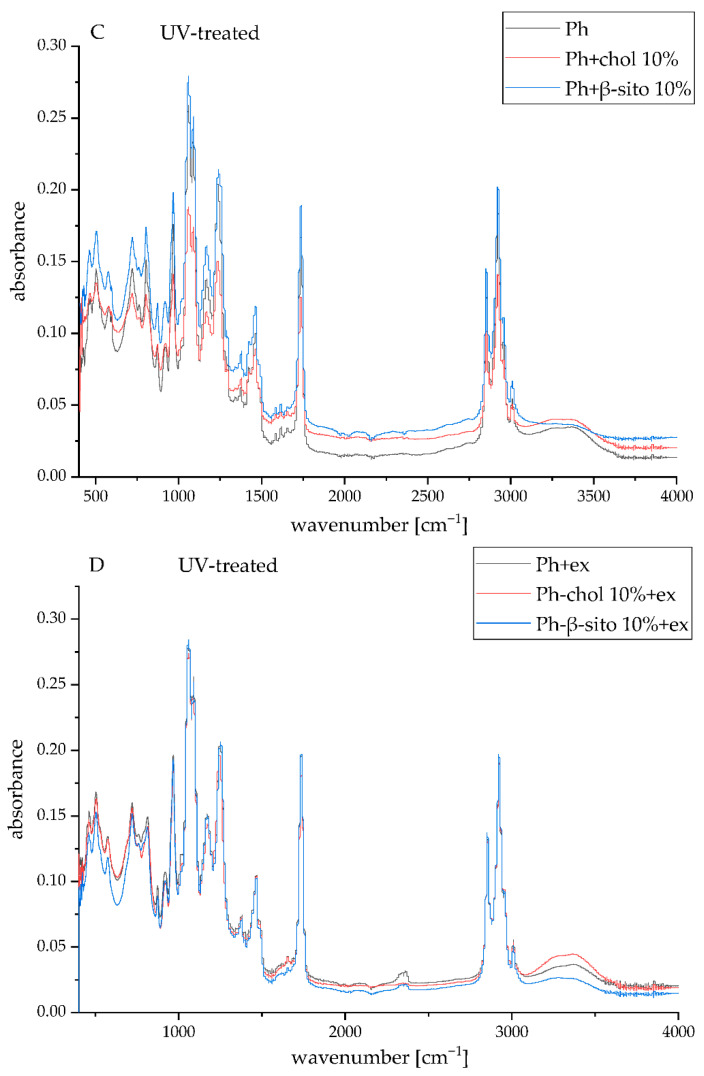
FT-IR spectra of non-treated plain (**A**) and extract-loaded liposomes (**B**), and UV-treated plain (**C**) and extract-loaded liposomes (**D**); liposomes containing 100% phospholipids (Ph), liposomes containing 90 mol% of Ph and 10 mol% of sterol, i.e., cholesterol or β-sitosterol (Ph+chol 10% and Ph+β-sito 10%, respectively); ex, extract.

FT-IR spectroscopy was used to investigate potential chemical alterations in phospholipids, sterols, and wild thyme extract components that may occur during liposome preparation. Additionally, this technique was also used to evaluate structural modifications in the lipid bilayer following physical treatments, including UV exposure. The corresponding spectral data of plain and extract-loaded liposomes (Ph, Ph+chol 10%, Ph+β-sito 10%, Ph+ex, Ph+chol 10%+ex, and Ph+β-sito 10%+ex) before and after UV irradiation are illustrated in Figure 3. FT-IR spectra of the initial compounds, including pure Phospholipon, lyophilized wild thyme extract, cholesterol, and β-sitosterol, are shown in Figure S2 (Supplementary Materials).

The FT-IR spectrum of the wild thyme extract exhibited a broad absorption band centered around 3250 cm^−1^, which is attributed to O–H stretching vibrations from hydroxyl, phenolic, and carboxylic functional groups (Figure S2). The peak detected at 2980 cm^−1^, along with those at 1450 cm^−1^ and 1328 cm^−1^, is associated with C–H stretching vibrations of methyl and methylene groups, C–H bending modes, and methyl group deformation, respectively [48]. A prominent peak at 1600 cm^−1^ corresponds to C=C stretching in aromatic and vinyl structures, while a series of absorptions between 1260–900 cm^−1^ are linked to asymmetric and symmetric stretching of C–O, C–O–C, and C–OH bonds typical of ethers, alcohols, phenols, and alkyl esters. Furthermore, deformation vibrations of =CH in aromatic and vinyl moieties are observed in the 760–720 cm^−1^ region [49,50]. These characteristic bands confirm the presence of key bioactive constituents in wild thyme extract, including flavonoids and phenolic acids [1,2].

The FT-IR spectra of pure Phospholipon (Figure S2) revealed peaks at around 3000 cm^−1^, 2925–2850 cm^−1^, and within the 1460–1335 cm^−1^ range, corresponding to =CH stretching, as well as -CH_2_ and -CH_3_ stretching and bending vibrations associated with the fatty acid chains, respectively [38]. Additional peaks at 1735 cm^−1^ and 1655 cm^−1^ are attributed to the stretching vibrations of ester C=O groups and overlapping O–H deformation with C=C stretching in unsaturated fatty acid residues, respectively. Vibrational bands in the 1260–965 cm^−1^ range arise from C–O–C and phosphate group stretching (P=O and P–O–C) [38]. Finally, bands at 875 cm^−1^ and in the 720–500 cm^−1^ region are linked to asymmetric P–O stretching and out-of-plane =C–H bending in *cis*-configured double bonds, respectively.

The FT-IR spectra of the liposomal formulations (untreated and UV-irradiated extract-loaded liposomes) demonstrated the presence of characteristic absorption bands attributed to phospholipids, suggesting successful integration of the extract into the liposomal spheres (Figure 3). The UV-treated samples showed no significant spectral changes detectable by FT-IR analysis, as illustrated in Figure 3.

### 3.6. Lipid Peroxidation of Developed Liposomes

**Figure 4:**
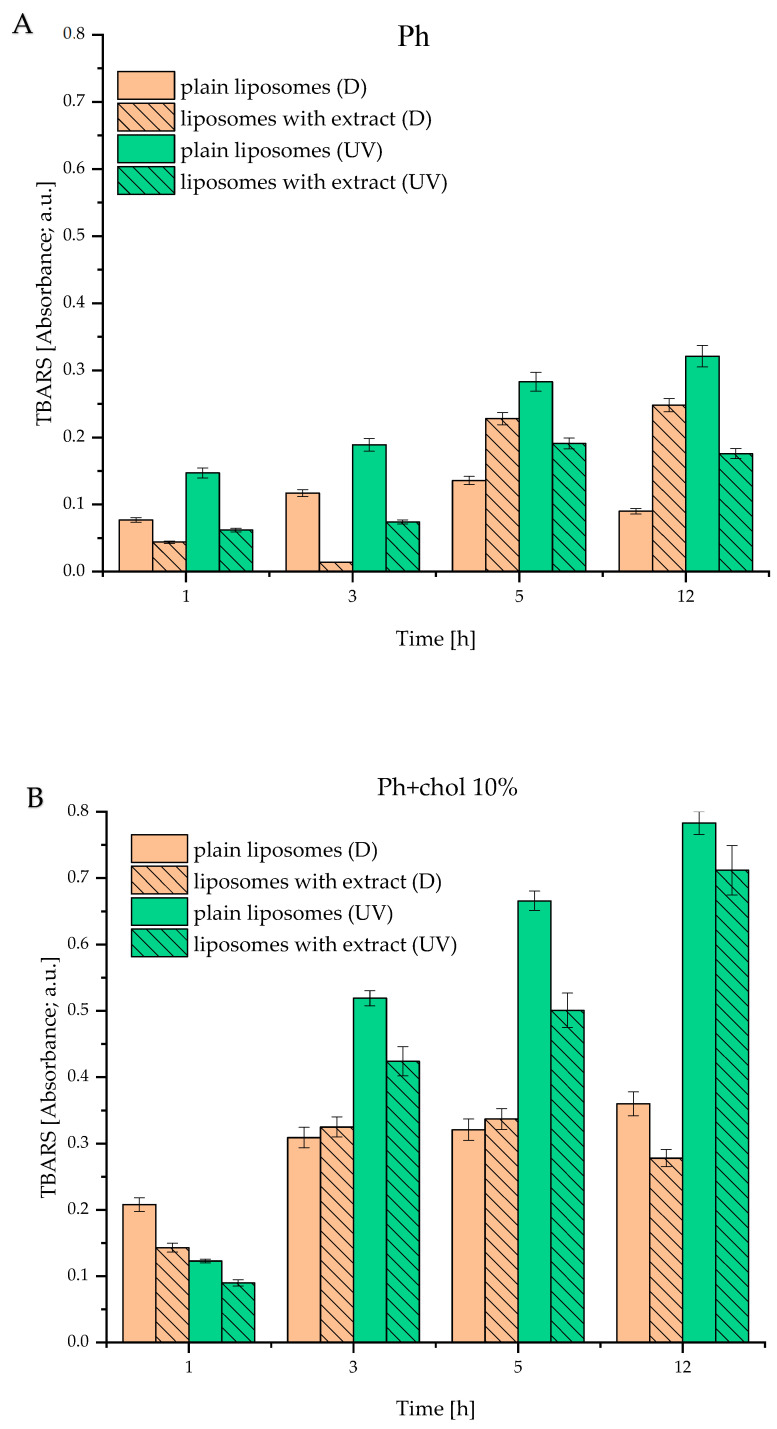

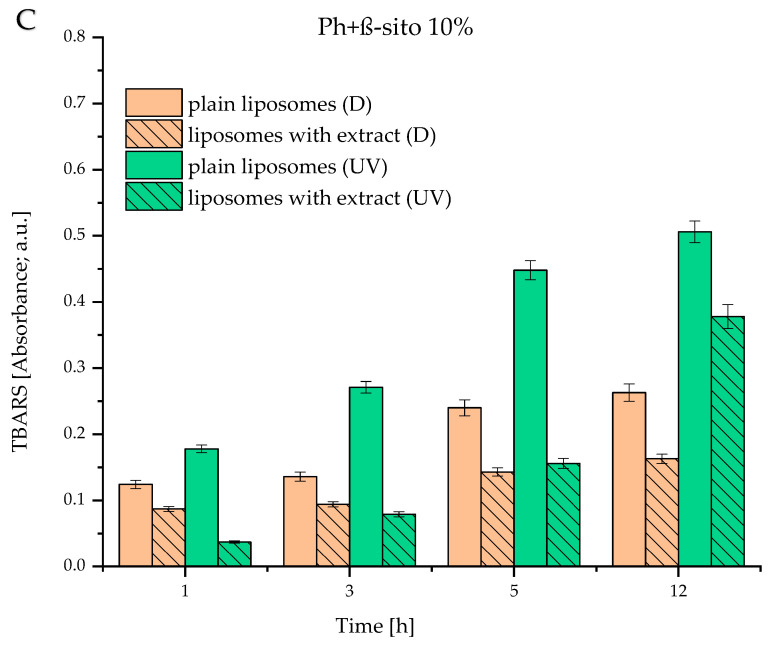
Effect of extract on liposomal oxidation (thiobarbituric acid reacting substances assay—TBARS; absorbance at 532 nm) under UV light (UV) and stored in the dark (D); (**A**) Ph, (**B**) Ph+chol 10%, and (**C**) Ph+β-sito 10% liposomes; liposomes containing 100% phospholipids (Ph), liposomes containing 90 mol% of Ph and 10 mol% of sterol, i.e., cholesterol or β-sitosterol (Ph+chol 10% and Ph+β-sito 10%, respectively).

The TBARS assay was employed to evaluate the extract’s potential to prevent or slow down the lipid peroxidation induced by UV light exposure. Namely, thiobarbituric acid reacts with the product of lipid hydroperoxide decomposition (malondialdehyde) [51]. This reaction produces a pink-colored complex, whose absorbance was measured spectrophotometrically at 532 nm. Results of the TBARS assay are presented in Figure 4 for Ph, Ph+chol 10%, and Ph+β-sito 10% liposomes. It is evident that liposomes without extract, when exposed to UV light, contained a higher amount of malondialdehyde, compared to those encapsulating wild thyme extract or control liposomes stored in the dark. For instance, after 5 h of irradiation, the extract reduced peroxidation by 1.5 to 3-fold, depending on the liposome sample.

These results demonstrate the antioxidant potential of wild thyme extract and suggest that it can protect liposomes prepared from commercial lipid mixtures against peroxidation. Literature data also support the protective role of polyphenols against lipid peroxidation [18,52]. On the other hand, Figure 4B,C illustrates that both cholesterol and β-sitosterol increased lipid peroxidation. This finding was expected, as our previous study revealed that lipid peroxidation depends on sterol content. Specifically, β-sitosterol and cholesterol exhibit a protective effect on lipid peroxidation only up to a certain concentration, beyond which the effect reverses [7]. A possible explanation for this phenomenon is that sterols themselves can undergo oxidation, thereby contributing to the overall peroxidation.

### 3.7. Polyphenol Release

**Figure 5:**
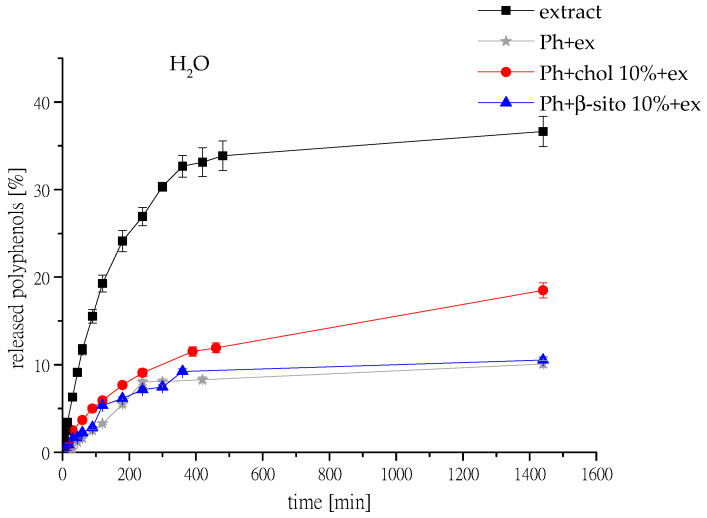
Kinetics of wild thyme polyphenol release from liquid wild thyme extract and from wild thyme extract-loaded phospholipid liposomes without sterols (Ph+ex), liposomes with 10 mol% of cholesterol (Ph+chol 10%+ex), and liposomes with 10 mol% of β-sitosterol (Ph+β-sito 10%+ex), observed in a Franz diffusion cell, in water medium at 25 °C; ex, extract.

**Figure 6:**
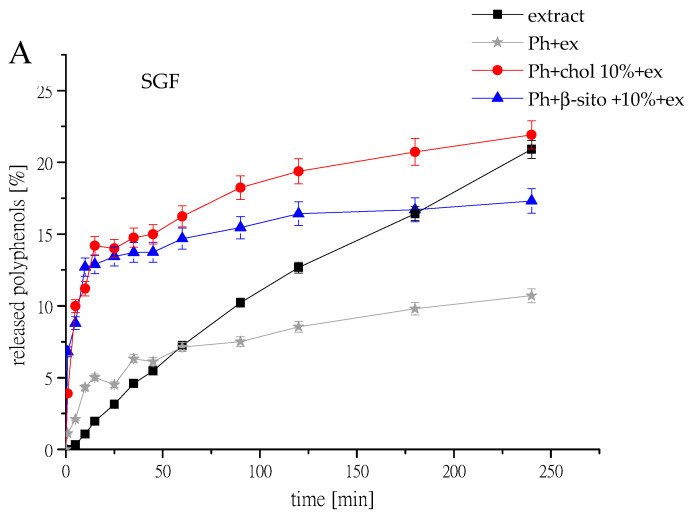

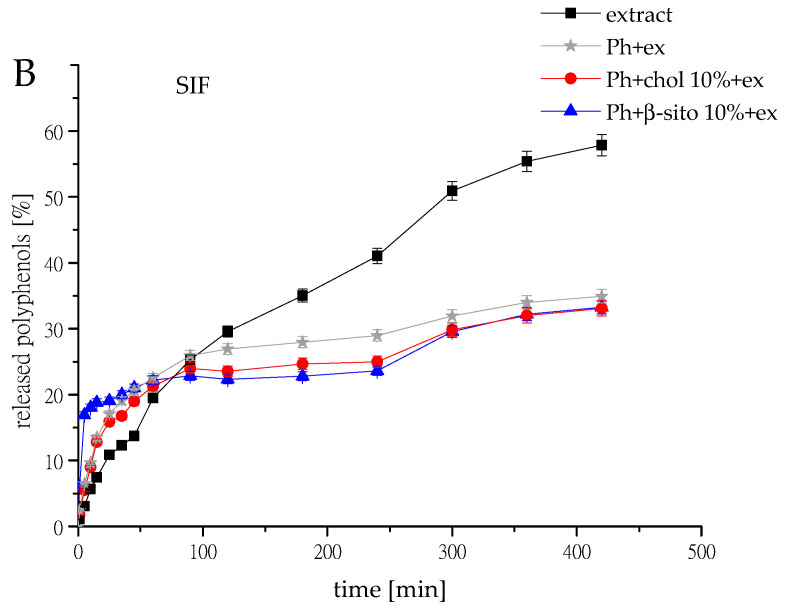
Kinetics of wild thyme polyphenol release from liquid wild thyme extract and extract loaded phospholipid liposomes without sterols (Ph+ex), liposomes with 10 mol% of cholesterol (Ph+chol 10%+ex) and liposomes with 10 mol% of β-sitosterol (Ph+β-sito 10%+ex), observed in Franz diffusion cell, in (**A**) simulated gastric fluid (SGF, pH 2.2) and (**B**) simulated intestinal fluid (SIF, pH 6.8) at 37 °C; ex, extract.

The release studies of polyphenols from selected samples (Ph+ex, Ph+chol 10%+ex, and Ph+β-sito 10%+ex) and pure extract were performed, and the mass transfer resistance of the liposomal membrane was calculated. The results are shown in Figure 5, where the released polyphenols were detected for 24 h in aqueous medium; release of polyphenols in simulated gastrointestinal fluids is presented in Figure 6. The release results obtained from the selected samples were compared with the release profile of the free unencapsulated extract solution. Diffusion coefficients (D) and diffusion resistance (R) of wild thyme extract and extract-loaded liposomes in water and simulated gastric and intestinal fluids are presented in Table 3.

**Table 3:** Diffusion coefficients (D) and diffusion resistance (R) of wild thyme extract and extract-loaded liposomes in water and simulated gastric and intestinal fluids.

Conditions	Sample	*D* [m^2^/s]	*R* [s/m]
Water, 25 °C	extract	1.41 · 10^−9^	2.17 · 10^6^
	Ph+ex *	2.68 · 10^−10^	1.14 · 10^7^
	Ph+chol 10%+ex	4.68 · 10^−10^	6.52 · 10^6^
	Ph+β-sito 10%+ex	2.01 · 10^−10^	1.52 · 10^7^
Simulated gastric fluid, 37 °C	extract	7.36 · 10^−10^	8.64 · 10^6^
	Ph+ex	2.74 · 10^−9^	7.42 · 10^5^
	Ph+chol 10%+ex	6.76 · 10^−9^	3.01 · 10^5^
	Ph+β-sito 10%+ex	9.57 · 10^−9^	2.13 · 10^5^
Simulated intestinal Fluid, 37 °C	extract	1.74 · 10^−9^	3.66 · 10^6^
	Ph+ex	5.96 · 10^−8^	3.42 · 10^4^
	Ph+chol 10%+ex	6.22 · 10^−9^	3.27 · 10^5^
	Ph+β-sito 10%+ex	2.50 · 10^−8^	8.18 · 10^4^

* liposomes containing 100% phospholipids (Ph), liposomes containing 90 mol% of Ph and 10 mol% of sterol, i.e., cholesterol or β-sitosterol (Ph+chol 10% and Ph+β-sito 10%, respectively); ex, extract.

As seen in Figure 5, the diffusion of polyphenols from the extract solution occurred rapidly. Within approximately 6 h, more than 30% of polyphenols had diffused from the solution, whereas for the same time interval, less than 10% of polyphenols were released from the liposomal formulations. In particular, the retention of polyphenols was slightly better in Ph+ex and Ph+β-sito 10%+ex compared to Ph+chol 10%+ex. As previously discussed, the extract possesses a higher amount of hydrophilic compounds, and literature data indicate that the presence of cholesterol tends to accelerate the release of such compounds [27]. It has already been demonstrated that cholesterol affects bilayer fluidity and, consequently, the release profile of active compounds [53]. Cholesterol controls the conformational and structural packing of phospholipids in lipid bilayers, and as a result, the rigidity of the fused ring structure stabilizes the straight chains in phospholipids. Similar results were reported by Maritim et al. [54], who observed that increasing cholesterol content led to a faster drug release rate.

In Figure 6, the release profiles of polyphenols from liposomes loaded with wild thyme by-product extract in SGF and SIF are presented. After 4 h of incubation in SGF, both samples, Ph+chol 10%+ex and Ph+β-sito 10%+ex, provided limited polyphenol release (17–22%), with a noticeable burst release in the first 20 min. This can be clarified by the fact that, under acidic conditions, liposomes tend to swell, facilitating the release of their core contents, even though they are structurally stable at low pH and provide protection for active compounds [55]. The slightly lower release observed for the Ph+β-sito 10%+ex compared to the Ph+chol 10%+ex may be attributed to a more favorable interaction of β-sitosterol molecules with phospholipids than of cholesterol molecules, resulting in a more stable liposomal formulation [7].

The same type of samples were exposed to SIF conditions for 6 h, and after that period, both Ph+β-sito 10%+ex and Ph+chol 10%+ex released about 32% of polyphenolic compounds. The leakage of the extract from liposomes could likely be attributed to the penetration of bile salts and pancreatin at this stage. The release trend observed was quite similar to that obtained in SGF conditions (particularly over the first 4 h), suggesting that the liposomes are highly stable nano-particulate systems, which inhibit the consumption of the extract to some extent in simulated gastrointestinal solution. This result is in agreement with the findings of Zhao et al. [56], who showed that the cumulative release of vitamin E from liposomes in SGF was around 15% higher than in SIF. Similarly, Wang et al. [57] reported that release profiles of fish oil from two kinds of liposomes in SIF were analogous to those in SGF, while Wang et al. [58] reported that after 3 h, the curcumin consumption was 21.82% in SGF and 27.32% in SIF. Literature reports various findings on the release rates of actives from liposomes in SGF and SIF. Some studies reported limited release of both hydrophobic and hydrophilic compounds in SGF, with a more substantial release in SIF [55,59,60], and others reported severe leakage (~80%) of bioactives from multivesicular liposomes in SGF compared to SIF [61]. These variations arise from several factors (such as the properties of the active compound, lipid type, preparation method, liposome size, SIF and SGF constituents, etc.), which together determine the specific outcomes. The incorporation of an additional gastro-resistant polymeric layer is strongly recommended to reduce release under gastric conditions and enhance the stability of liposomes [62].

## 4. Conclusions

This study investigated the impact of cholesterol and β-sitosterol on the physicochemical properties, EE, stability, thermal properties, and delivery profile of liposomes containing polyphenol-rich wild thyme by-product extract. The results demonstrate that incorporating cholesterol and β-sitosterol into phospholipid-based liposomes significantly affects their EE, particle size, membrane stability, thermal characteristics, and release kinetics. The highest encapsulation of wild thyme polyphenols was achieved with 10 mol% sterol inclusion, while higher concentrations resulted in reduced EE due to steric hindrance and increased membrane permeability. β-sitosterol was particularly effective in enhancing liposome stability, as evidenced by higher absolute zeta potential values and robust behavior under UV exposure and lyophilization. Furthermore, thermal analysis confirmed that both sterols and polyphenols interact with lipid membranes, influencing phase transition behavior. FT-IR analysis verified the incorporation of key polyphenolic constituents from the extract into liposomes. The extract exhibited strong antioxidant capacity, significantly reducing lipid peroxidation under UV irradiation, particularly in formulations without additional sterols. Controlled release behavior was observed in both SGF and SIF, with β-sitosterol-containing liposomes demonstrating slightly enhanced retention. These results highlight the potential of wild thyme extract-loaded liposomes to protect bioactive compounds from degradation and to modulate their release in gastrointestinal conditions. Moreover, the results emphasize the critical role of sterol content optimization in balancing membrane integrity and resistance to oxidative stress. This work contributes to the growing interest in plant-based antioxidants and lipid nanocarriers for functional food applications, offering promising opportunities to enhance the efficacy and stability of natural bioactives in oral delivery systems. Collectively, these findings suggest that sterol-enriched liposomes, particularly those containing phytosterols like β-sitosterol, are promising vehicles for delivering plant-derived bioactives and enhancing their stability in food systems. Future experiments will include the potential impact of cryoprotectants on membrane integrity and stability, as well as the investigation of the biological potential of developed liposomes.

## Figures and Tables

**Table 1:** Efficiency of encapsulation (EE) of wild thyme extract in the liposomes (the values obtained immediately after the liposomal preparation, UV irradiation, and lyophilization).

Samples	EE [%]		
	Non-Treated	UV	Lyophilized
Ph+ex *	89.4 ± 0.8 ^c,1,^*	87.8 ± 1.0 ^d,1^	88.3 ± 0.1 ^d,1^
Ph+chol 10%+ex	94.4 ± 0.4 ^a,1^	94.8 ± 0.7 ^a,1^	94.4 ± 1.1 ^a,1^
Ph+chol 20%+ex	91.2 ± 0.8 ^bc,1^	90.1 ± 0.4 ^c,1^	91.7 ± 0.2 ^b,1^
Ph+chol 30%+ex	82.2 ± 0.8 ^d,1^	81.8 ± 0.6 ^e,1^	80.7 ± 1.5 ^e,1^
Ph+β-sito 10%+ex	92.8 ± 0.9 ^b,1^	93.0 ± 0.6 ^b,1^	92.2 ± 0.3 ^b,1^
Ph+β-sito 20%+ex	88.9 ± 0.7 ^c,1^	90.1 ± 0.9 ^c,1^	90.0 ± 1.3 ^c,1^
Ph+β-sito 30%+ex	79.1 ± 0.9 ^e,1^	77.7 ± 1.0 ^f,1^	78.4 ± 1.0 ^e,1^

* liposomes containing 100% phospholipids (Ph), liposomes containing 90–70 mol% of Ph and 10–30 mol% of sterol, i.e., cholesterol or β-sitosterol (Ph+chol and Ph+β-sito, respectively); values with the same letter (a–f) in each column (related to the potential differences between various liposomal populations) and the same number in each row (related to the potential differences between the same liposomal population exposed to different post-preparation treatment or modification) showed no statistically significant difference (*p* > 0.05; n = 3; analysis of variance, Duncan’s post hoc test); ex, extract.

**Table 2:** The transition temperature and enthalpy change of Phospholipon, lyophilized wild thyme extract, plain, and extract-loaded liposomes.

Sample	Temperature (°C)			ΔH (J/g)
	Onset	Peak	Offset	
Phospholipon *	162.4	165.8	170.9	110.1
Extract	50.9	63.6	96.7	65.1
Ph	125.1	131.1	135.1	42.7
Ph+ex	117.7	122.4	124.9	0.9
Ph+chol 10%	72.2	83	92.4	55.9
Ph+chol 10%+ex	71.4	73.1	78.99	3.28
Ph+β-sito 10%	51.7	53.9	63	347.7
	106.9	109.3	113.4	58.6
Ph+β-sito 10%+ex	71.2	60.7	82.1	813.7
	103	107.8	113.4	459.5

* Phospholipon (commercial mixture of phospholipids used for the liposome preparation); liposomes containing 100% phospholipids (Ph), liposomes containing 90 mol% of Ph and 10 mol% of sterol, i.e., cholesterol or β-sitosterol (Ph+chol 10% and Ph+β-sito 10%, respectively); ex, extract.

## Data Availability

The original contributions presented in this study are included in the article and Supplementary Material. Further inquiries can be directed to the corresponding author.

## Supplementary Materials

The following supporting information can be downloaded at: https://www.mdpi.com/article/10.3390/foods14152626/s1, Figure S1: The vesicle size (A,B), polydispersity index (C,D), and zeta potential (E,F) of plain liposomes and wild thyme extract-loaded liposomes, respectively, measured during 60 days of refrigerated storage; liposomes containing 100% phospholipids (Ph), liposomes containing 90–70 mol% of Ph and 10–30 mol% of sterol, i.e., cholesterol or β-sitosterol (Ph+chol and Ph+β-sito, respectively); ex, extract; Figure S2: FT-IR spectra of pure Phospholipon (commercial mixture of phospholipids used for the liposome preparation), lyophilized wild thyme extract, cholesterol, and β-sitosterol.

## Abbreviations

The following abbreviations are used in this manuscript:

ANOVA	Analysis of Variance
EE	Encapsulation Efficiency
DSC	Differential Scanning Calorimetry
PCS	Photon Correlation Spectroscopy
PDI	Polydispersity Index
Ph	Phospholipids
SGF	Simulated Gastric Fluid
SIF	Simulated Intestinal Fluid
TPC	Total Polyphenol Content
