# Supplementary material for: Liposome-Based Encapsulation of Extract from Wild Thyme (Thymus serpyllum L.) Tea Processing Residues for Delivery of Polyphenols
Source: Foods. 2025 Jul 26;14(15):2626. doi: 10.3390/foods14152626 (PMC12346670; doi:10.3390/foods14152626)
Supplement: Supplementary file 1 [file foods-14-02626-s001.zip › foods-3734992-supplementary.pdf]

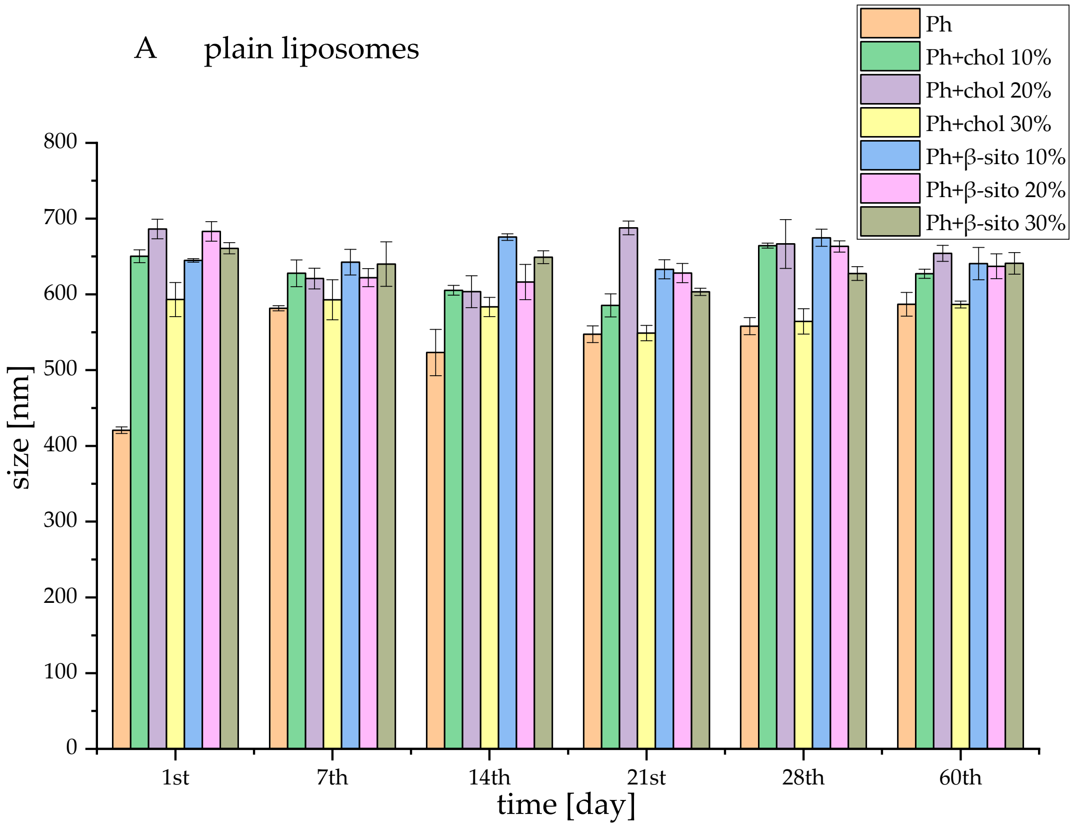
A plain liposomes
Ph
Ph+chol 10%
Ph+chol 20%
Ph+chol 30%
Ph+β-sito 10%
Ph+β-sito 20%
Ph+β-sito 30%
size [nm]
0
100
200
300
400
500
600
700
800
1st
7th
14th
21st
28th
60th
time [day]

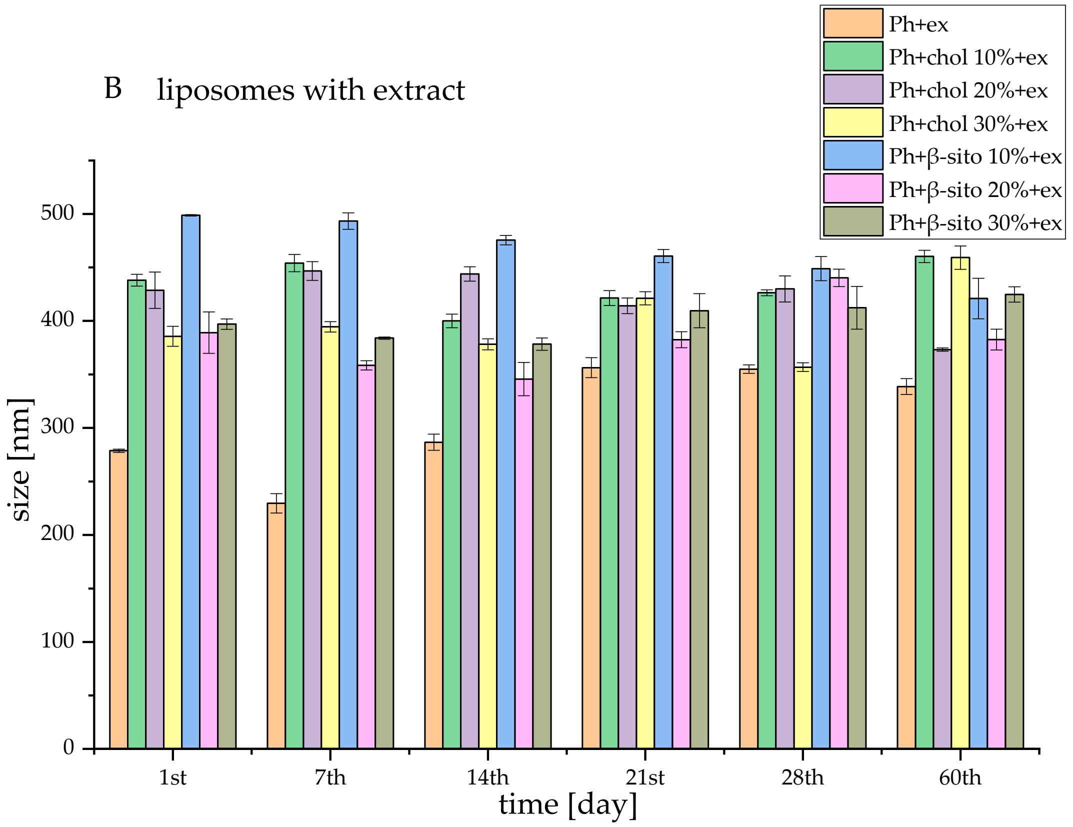
B liposomes with extract
Ph+ex
Ph+chol 10%+ex
Ph+chol 20%+ex
Ph+chol 30%+ex
Ph+β-sito 10%+ex
Ph+β-sito 20%+ex
Ph+β-sito 30%+ex
size [nm]
0
100
200
300
400
500
1st
7th
14th
21st
28th
60th
time [day]

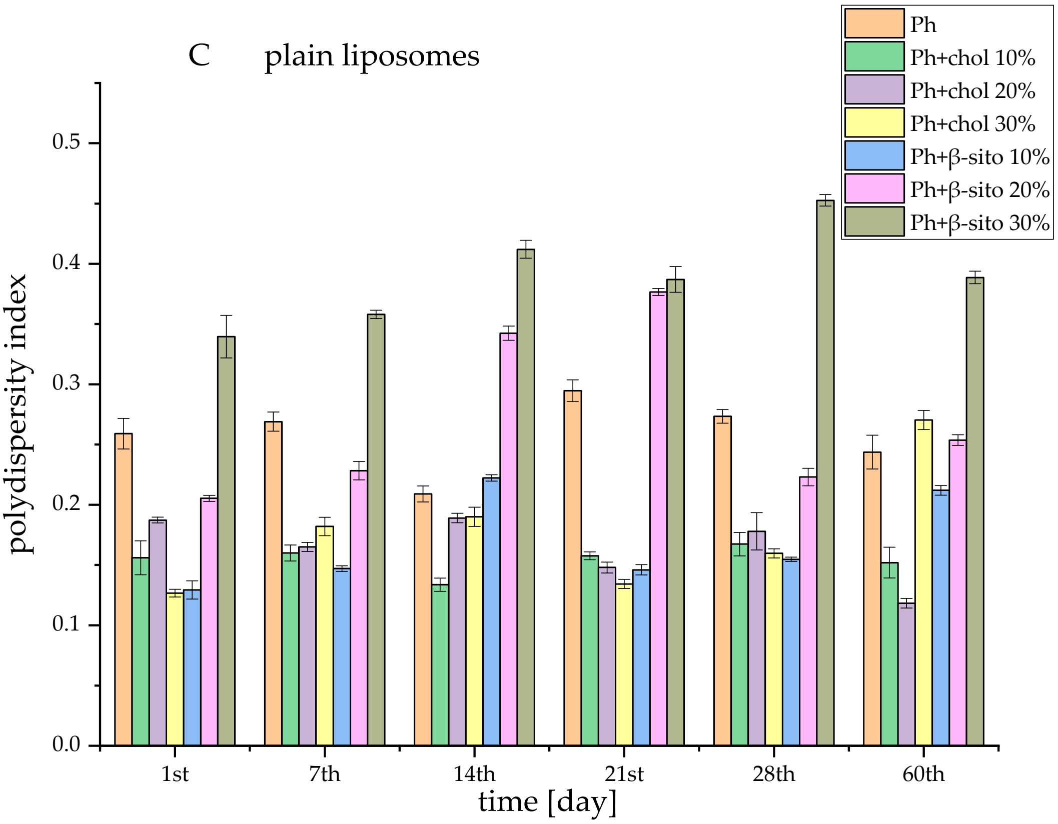
C plain liposomes
polydispersity index
time [day]
1st
7th
14th
21st
28th
60th
0.0
0.1
0.2
0.3
0.4
0.5
Ph
Ph+chol 10%
Ph+chol 20%
Ph+chol 30%
Ph+β-sito 10%
Ph+β-sito 20%
Ph+β-sito 30%

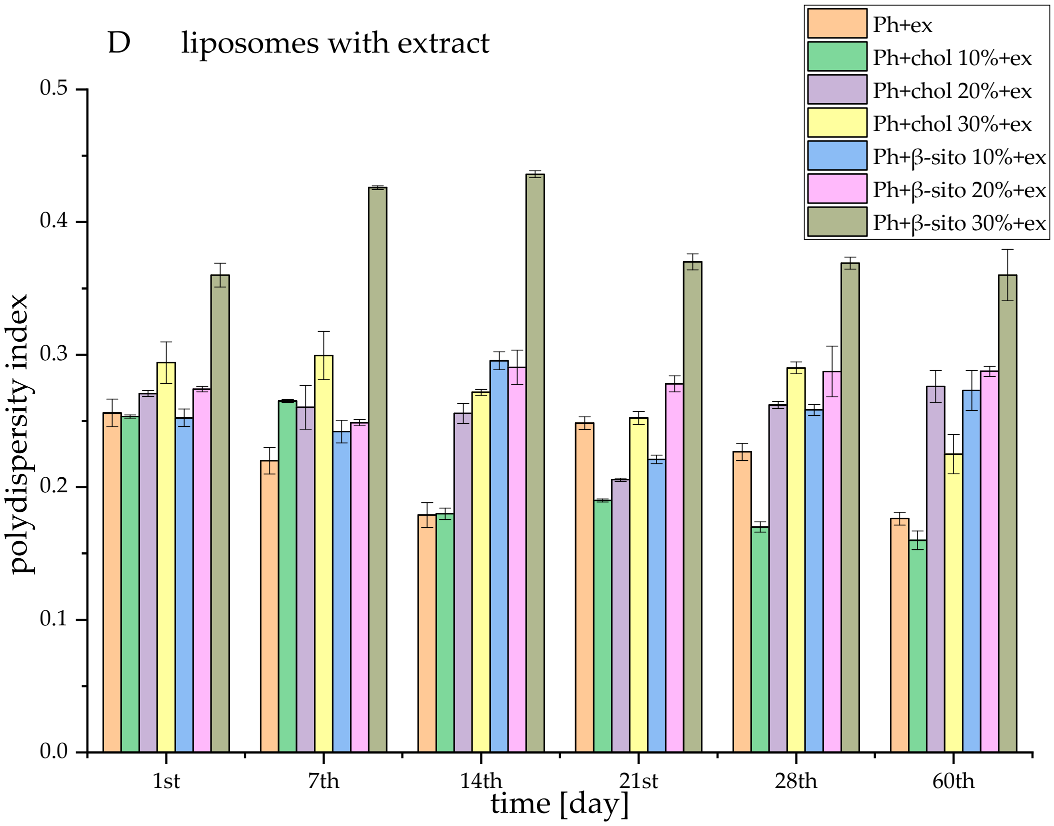
D liposomes with extract
polydispersity index
time [day]
1st
7th
14th
21st
28th
60th
0.0
0.1
0.2
0.3
0.4
0.5
Ph+ex
Ph+chol 10%+ex
Ph+chol 20%+ex
Ph+chol 30%+ex
Ph+β-sito 10%+ex
Ph+β-sito 20%+ex
Ph+β-sito 30%+ex

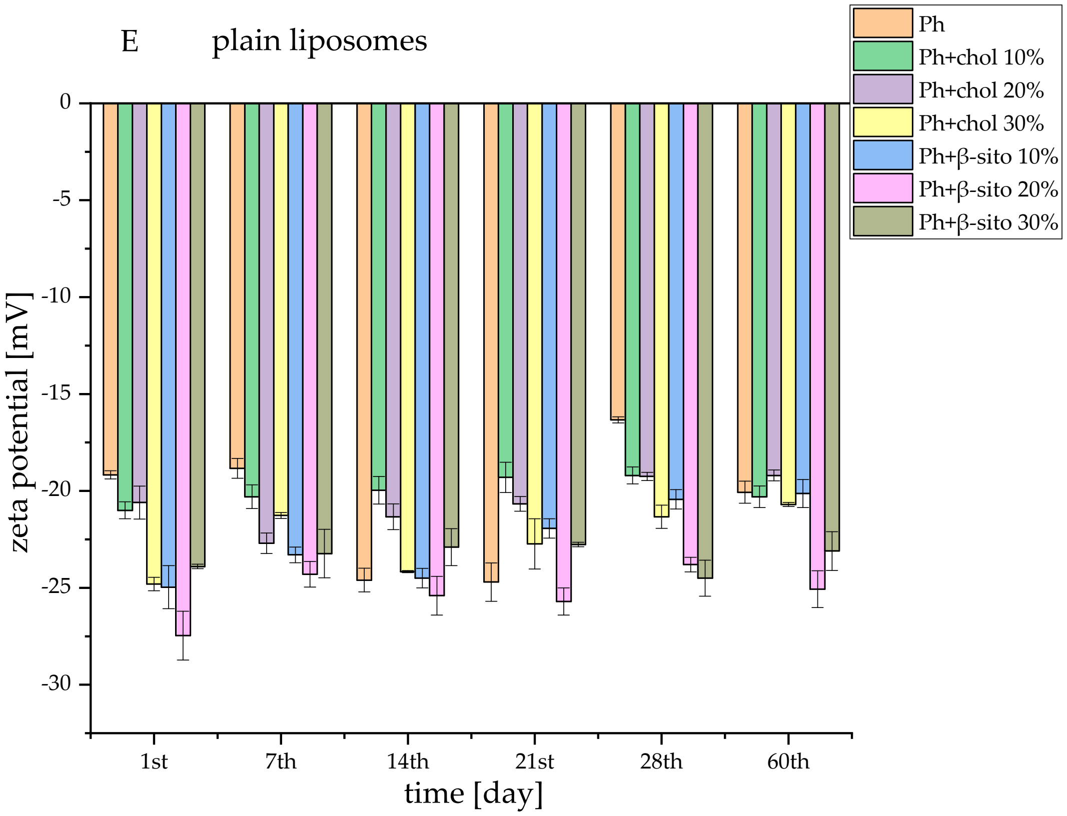

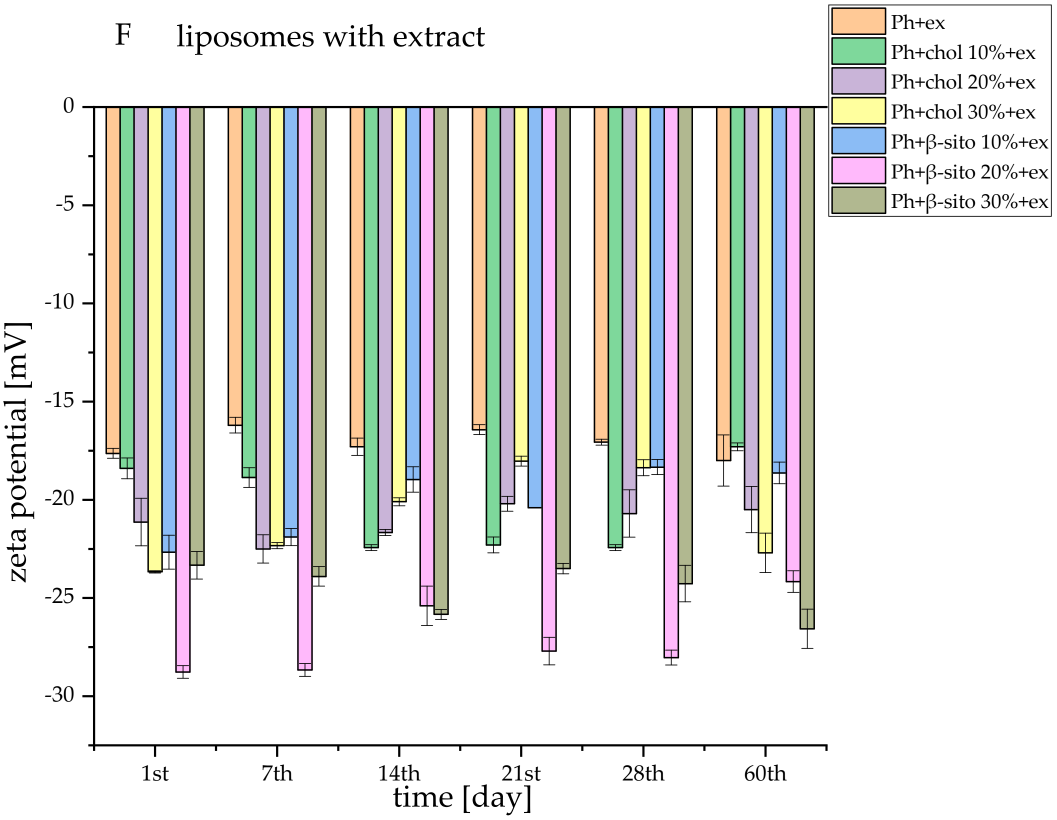

Figure S1. The vesicle size (A and B), polydispersity index (C and D), and zeta potential (E and F) of plain liposomes and wild thyme extract-loaded liposomes, respectively, measured during 60 days of refrigerated storage; liposomes containing 100% phospholipids (Ph), liposomes containing 90-70 mol% of Ph and 10-30 mol% of sterol, *i.e.*, cholesterol or β-sitosterol (Ph+chol and Ph+β-sito, respectively); ex, extract.

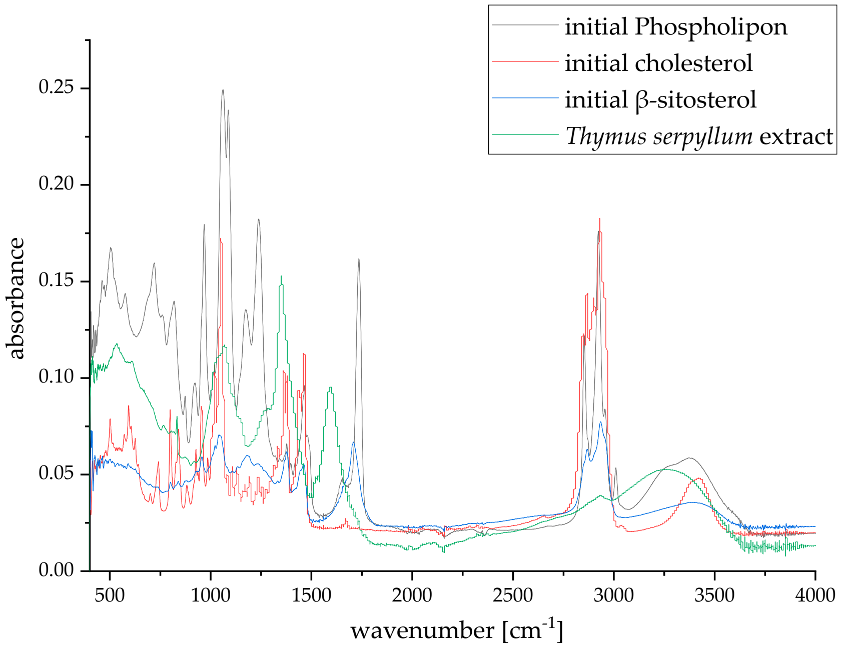

Figure S2. FT-IR spectra of pure Phospholipon (commercial mixture of phospholipids used for the liposome preparation), lyophilized wild thyme extract, cholesterol, and β-sitosterol.
